# Focused ion beam-induced platinum deposition with a low-temperature cesium ion source

**DOI:** 10.3762/bjnano.16.69

**Published:** 2025-06-16

**Authors:** Thomas Henning Loeber, Bert Laegel, Meltem Sezen, Feray Bakan Misirlioglu, Edgar J D Vredenbregt, Yang Li

**Affiliations:** 1 Nano Structuring Center (NSC), Rheinland-Pfälzische Technische Universität Kaiserslautern-Landau (RPTU), P.O. Box 3049, D-67653, Kaiserslautern, Germany; 2 Sabanci University Nanotechnology Research and Application Center (SUNUM), 34956, Istanbul, Turkey; 3 Department of Applied Physics, Eindhoven University of Technology, P.O. Box 513, 5600 MB Eindhoven, The Netherlands

**Keywords:** cesium ion source, cold atom ion source, focused ion beam (FIB), FIB-induced deposition (FIBID)

## Abstract

In addition to precise milling, the deposition of material at a specific location on a sample surface is a frequently used process of focused ion beam (FIB) systems. Here, we report on the deposition of platinum (Pt) with a new kind of cesium (Cs) FIB, in which the cesium ions are produced by a low-temperature ion source. Platinum was deposited at different acceleration voltages and ion beam currents. Deposition rate, material composition, and electrical resistivity were examined and compared with layers deposited at comparable settings with a standard gallium (Ga) FIB. The deposition rate is found to depend linearly on the current density. The rate is comparable for Cs^+^ and Ga^+^ under similar conditions, but the deposit has lower Pt content for Cs^+^. The electrical resistivity of the deposit is found to be higher for Cs^+^ than for Ga^+^ and decreasing with increasing acceleration voltage.

## Introduction

The deposition of material at a certain spot on a sample surface is a powerful and useful feature of focused ion beam (FIB) systems. At first, the deposition was used for circuit editing and as a protection layer before milling. Nowadays, the process is more far-reaching, and three-dimensional magnetic or superconductive structures can be created [1–4]. Also, specific mechanical structures on atomic force microscopy (AFM) cantilevers can be made [5–6]. In the literature, four mechanisms are used to explain the complex process of focused ion beam-induced deposition (FIBID) [5,7]; the major role is played by the primary ion beam, together with a thermal heat spike, excited surface atoms (ESA), or secondary electrons (SE). According to Hlawacek et al. [8], the number of ESA is proportional to the nuclear stopping power, so for heavier ions this mechanism dominates the deposition. The exact order, however, of which mechanism contributes how much to the deposition, for example, for cesium (Cs) ions, is beyond the scope of this paper since FIBID is rather complex and depends on a variety of parameters. Besides beam parameters such as acceleration voltage, beam current, ion dose, dwell time, and refresh time, precursor material and substrate have an influence on the effective deposition rate. Gallium (Ga^+^) and helium (He^+^) are the most often utilized ion species for FIBID [1–2,5].

Besides these standard FIB systems, new kinds of laser-cooled ion sources have been developed in the last few years. One strength of these ion sources, which are based on laser-cooled atoms, is that many elements unavailable with conventional sources can be used. At least 27 elements, including metals and non-metals, have successfully been laser-cooled [9]. Among these elements, rubidium (Rb) and Cs are more advanced with respect to source development because of their relatively low requirements regarding the cooling laser. Milling [10] as well as induced deposition of platinum (Pt) [11] and tungsten (W) [12] have been studied for a prototype FIB with an ultracold Rb^+^source.

Further, a Cs^+^ laser-cooled ion source (LoTIS) has been developed and characterized [13–14]. Like the Rb^+^ source, the Cs^+^ LoTIS has also been incorporated in a standard Ga FIB column. Loeber et al. have shown several advantages of the cold Cs^+^ FIB in imaging [15] and milling [16] applications over standard Ga^+^ FIBs. Compared to a standard Ga^+^ FIB, the Cs^+^ FIB can produce images with higher resolution and a larger depth of focus. Furthermore, the material contrast is greater in images acquired with Cs^+^ compared to images acquired with Ga^+^. For milling applications, Cs^+^ can deliver more evenly etched pattern floors than Ga^+^. With these benefits in applications established, microscopy and induced deposition studies help to establish more properties of the Cs^+^ FIB. Given the similar sources and FIB column designs of Cs^+^ and Rb^+^ FIBs, it is useful to compare applications with these FIBs to understand the relative merits of ion sources based on alkali metals. Also, Cs^+^ is a preferred species of ions over Ga^+^ for secondary ion mass spectroscopy (SIMS) applications because Cs^+^ can induce higher secondary ion yields for several elements such as carbon (C), oxygen (O), and hydrogen [17–19], which provides higher signal-to-noise ratios for SIMS analysis. A disadvantage of using Cs^+^ is a possible surface modification [20–22]. One aspect of this paper is to show whether it is at all possible to deposit Pt with Cs^+^ ions or whether surface modifications dominate [10].

This work presents FIBID experiments using a Cs^+^ FIB in comparison to results of layer deposition induced by Rb^+^ and Ga^+^. Pt was deposited at different acceleration voltages and ion beam currents to evaluate the deposition rate and the electrical resistivity of the layers. To measure the grain structure as well as the material composition using energy-dispersive X-ray spectroscopy (EDS), lamellas for transmission electron microscopy (TEM) were prepared.

## Experimental

The Ga^+^ FIB is a ThermoFisher Helios NanoLab 650 and uses a gas injection system (GIS). ZeroK NanoTech Corporation has created commercially available Cs^+^ FIB systems based on standard ion columns from ThermoFisher [23]. Both the Cs^+^ and the Rb^+^ FIB are equipped with a standard Pt GIS. The same precursor trimethylplatinum, C_5_H_4_CH_3_Pt(CH_3_)_3_, was used for all FIBID-Pt experiments. The precursor was heated to 40 °C to create a gas flow through the GIS nozzle, with the exit of the nozzle kept about 100 μm above the sample surface. The chamber pressure of the Cs^+^ and the Ga^+^ FIB was about 5 × 10^−7^ mbar before deposition and 8 × 10^−6^ mbar during deposition. For Pt deposition, a beam step size of −150% of the beam diameter was used with an upper limit of 200 nm for the Cs FIBID to avoid, for example, any inhomogeneous ripple structures. The dwell time was always 200 ns.

For growth rate characterization, Pt layers with a length of 20 μm and a width of 1 μm were deposited on silicon (Si). The ion beam currents were changed, while the pattern size was kept constant. With the Cs^+^ FIB, ion currents from 16 to 285 pA were used, so the current densities were between 0.9 and 14.2 pA·μm^−2^. The overall deposition time was kept constant at 2:30 min, and the ion dose was changed from 128 to 2138 pC·μm^−2^. The ions were accelerated with voltages of 2, 5, 8, and 16 kV, while the measured ion beam diameter changed with voltage and current from 32 to 445 nm.

With the Ga^+^ FIB, patterns were deposited at ion beam currents ranging from 17 to 396 pA with current densities between 0.7 and 19.8 pA·μm^−2^. With a deposition time of 2:30 min, the ion dose is between 105 and 2970 pC·μm^−2^. The diameter of the ion beam is specified as ranging from 10 to 159 nm, changing with acceleration voltage (5, 8, 16, and 30 kV) and ion beam current. The actual thickness of each layer was measured with a standard cross section using the Ga^+^ FIB. All scanning electron microscopy (SEM) images were taken with the NanoLab 650 dual beam system. All parameters of the FIBID for the growth rate measurements can be seen below in Table 2 in the Appendix section.

The electrical resistivity of Ga^+^ and Cs^+^ FIBID-Pt was measured via the Cr-on-glass standards mentioned in [11] using the sample design displayed below in Figure 8a. The pattern size was 35 μm by 1.5 μm. The deposition time was varied with the ion beam current and the acceleration voltage to achieve a constant layer thickness of approximately 1000 nm. With the Ga^+^ FIB, ion beam currents between 30 and 630 pA were used, so the current densities ranged from 0.9 to 8.4 pA·μm^−2^. The deposition time was calculated between 4:42 and 24:24 min with ion doses ranging from 1254 to 2276 pC·μm^−2^. With acceleration voltages of 8, 16, and 30 kV and the used ion beam currents, the beam diameter changes from 32 to 208 nm.

With the Cs^+^ FIB, ion beam currents between 13 and 440 pA, corresponding to current densities between 0.4 and 8 pA·μm^−2^, were utilized. The deposition time was between 3:05 and 16:11 min, and the total ion dose ranged between 717 and 1921 pC·μm^−2^. Using acceleration voltages of 2, 5, 8, and 16 kV, the ion beam diameter changed from 25 to 460 nm. All deposition parameters are shown in Table 1. To calculate the resistivity of the deposits, the NanoLab 650 dual beam system was used to determine the length and the cross section of each deposited layer.

**Table 1:** The deposition parameters are shown for the growth rate and the electrical resistivity measurements for both ion species.

		Acceleration voltage [kV]		Ion current [pA]		Beam size [nm]		Deposit area [μm^2^]	Current density [pA·μm^−2^]		Deposition time [min:s]		Ion dose [pC·μm^−2^]	
Deposition for	Ion	from	to	from	to	from	to		from	to	from	to	from	to

growth rate	Ga	5	30	17	396	10	159	20	0.7	19.8	2:30		105	2970
	Cs	2	16	16	285	32	445	20	0.9	14.2	2:30		128	2138
resistivity	Ga	8	30	30	630	32	208	52.5	0.9	8.4	4:42	24:24	1254	2276
	Cs	2	16	13	440	25	460	52.5	0.4	8	3:05	16:11	717	1921

The TEM lamellas were prepared with a JEOL JIB 4601F FIB-SEM MultiBeam system. The sample characterization in terms of elemental composition and structure was done with a JEOL ARM 200F (S)TEM system equipped with an Oxford EDS detector.

## Results and Discussion

### Deposit surface

First, to reveal possible surface modifications, Pt layers were deposited with Cs^+^ ions at 16, 8, 5, and 2 kV with a current density of 6 pA·μm^−2^ . Before deposition, a small part of the Si substrate was gently milled with the Cs^+^ FIB at 16 kV, such that any native oxide and other contaminations were completely removed at this location. With that, the influence of oxygen molecules on the deposited layer can be excluded. The layers, which have a length of 40 μm and a width 1 μm, were deposited across the boundary between the bare Si and the Si with its native oxide intact.

**Figure 1:**
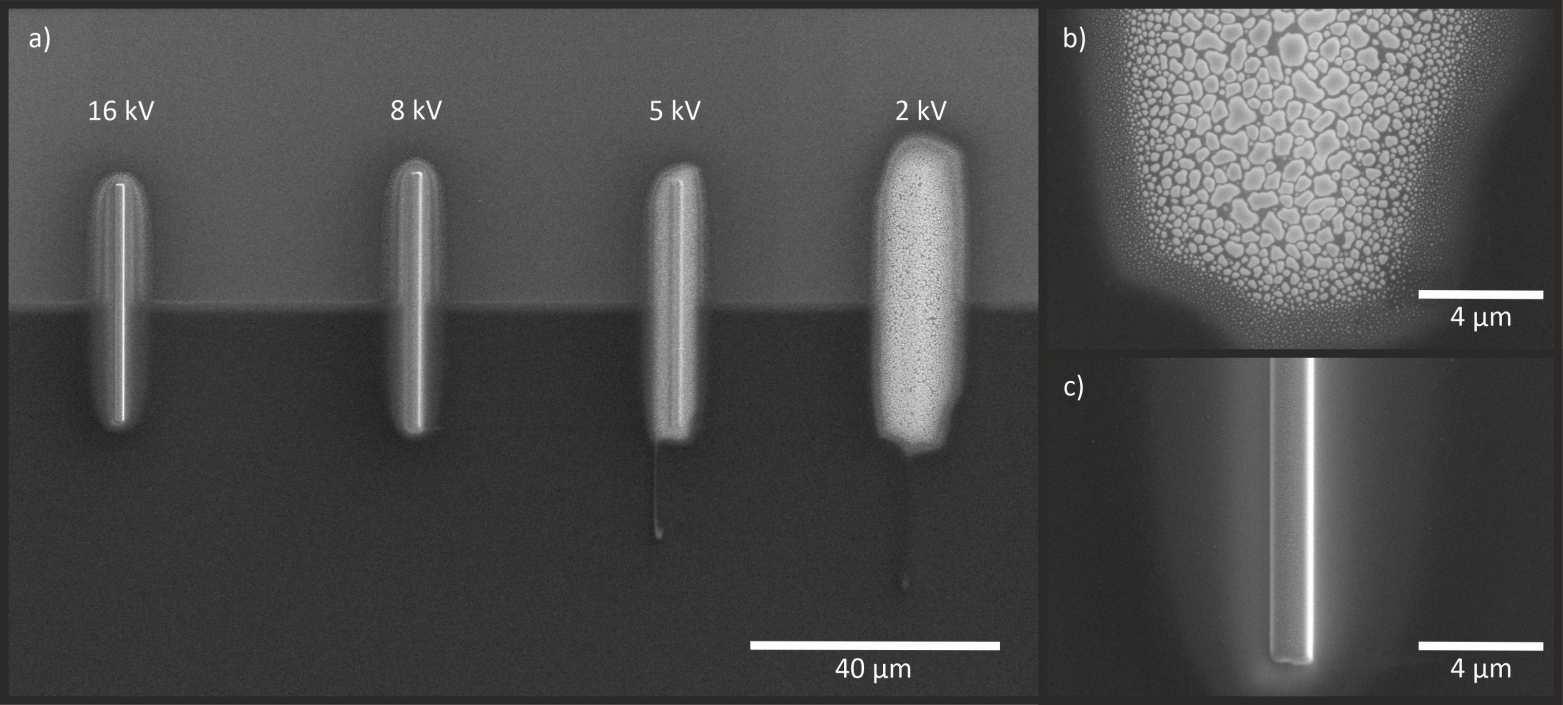
(a) SEM images of Pt deposited with Cs^+^ ions at 16, 8, 5, and 2 kV on Si. The upper part of the image appears brighter because the native oxide of Si was removed before the deposition. The amount of surface bubbles increases with decreasing acceleration voltage, while no difference between Si and Si with oxide can be observed. (b) FIBID-Pt on Si with Cs^+^ ions at 2 kV showing the highest amount of bubbles. (c) Pt deposited at 16 kV with no bubbles.

A visual inspection with the SEM reveals surface bubbles on layers deposited at 2 and 5 kV (as shown in Figure 1a). No significant differences can be seen in the size or density of the bubbles when comparing depositions on Si (upper part) and Si with the native oxide layer (lower part). Similar to Rb^+^ FIBID-Pt discussed in [11], these bubbles appear after exposure of the samples to air (during the short transfer from the Cs^+^ FIB to the Ga^+^ FIB) and are more numerous and larger at 2 kV than at 5 kV. At higher acceleration voltages, bubbles are not observed (see Figure 1b,c).

**Figure 2:**
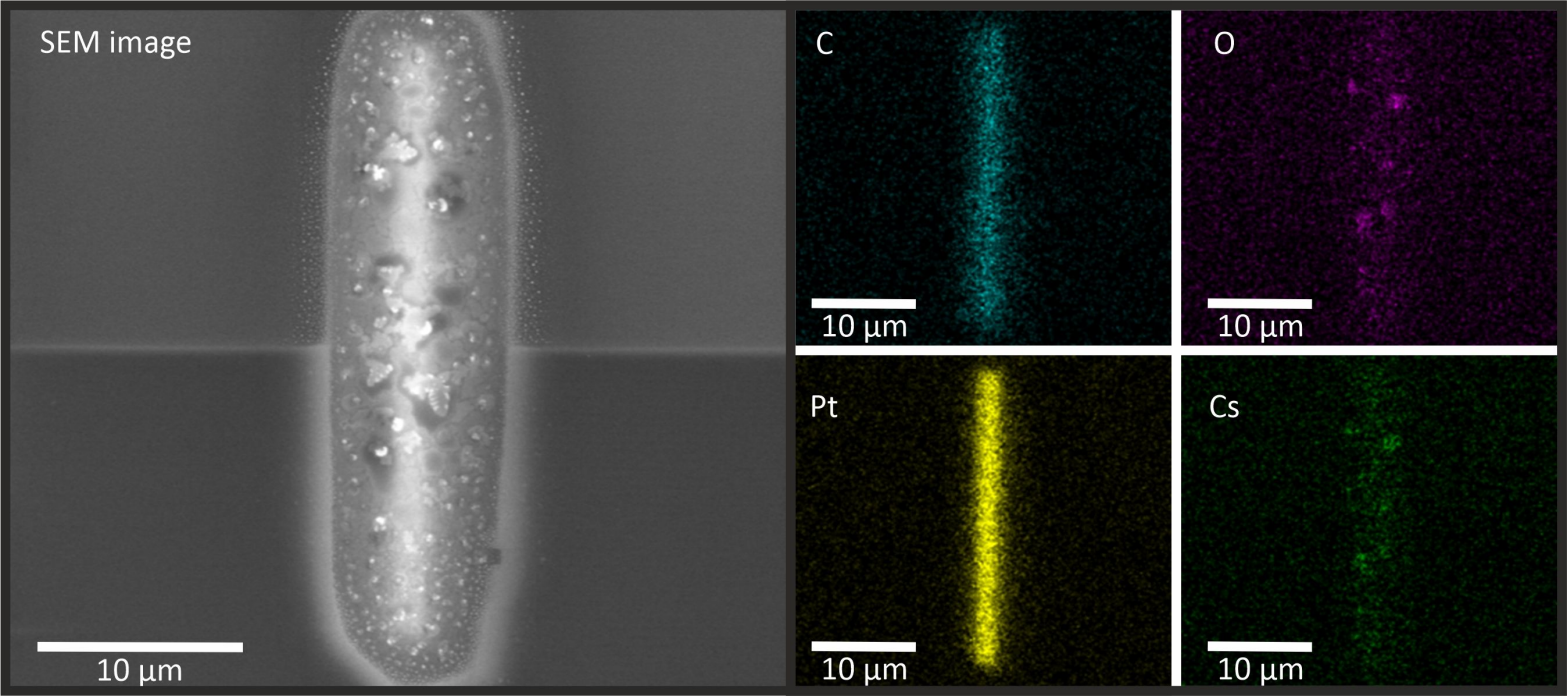
SEM and EDS analysis of a Pt layer deposited at 2 kV with Cs^+^ ions. The SEM image shows an overview of the layer, while the other images depict the individual material distribution. The actual shape of the layer can be seen in the images of the Pt and C concentration, while the bubbles mainly consist of Cs and O. While the highest concentration of Pt and C can be found within the actual shape, the Cs and O distribution correspond to the bubble area in the SEM image.

EDS measurements displayed in Figure 2 show that these bubbles mainly consist of Cs and O. This is also consistent with the observations reported in [11] on surface bubbles in Rb^+^ FIBID-Pt.

**Figure 3:**
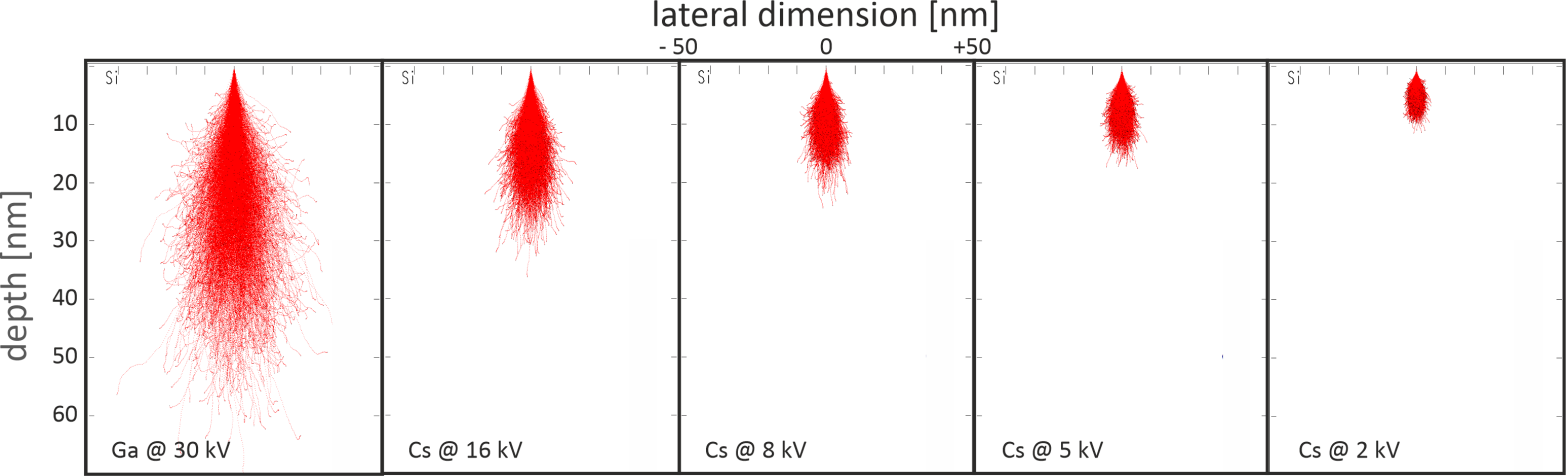
SRIM simulation showing the penetration of Ga^+^ and Cs^+^ ions into a Si substrate at different acceleration voltages. Cs^+^ ions at a voltage of 2 and 5 kV remain much closer to the surface and can react more with the Pt precursor elements on the surface.

The proposed mechanism for the formation of these bubbles is that elements of the Pt precursor trap the primary alkali metal ions when the chemical bonds of the precursor are not completely broken. With higher ion beam voltage, more bonds are broken and volatile elements including Cs^+^ ions are sputtered from the surface and pumped away by the vacuum system. Also, Cs^+^ ions with higher energies penetrate deeper into the material (see the “Stopping and Range of Ions in Matter” (SRIM) [24] simulations in Figure 3) and react less with the elements on the surface. Therefore, the surface bubbles diminish with higher acceleration voltages. This is why only 2 and 5 kV Cs^+^ depositions lead to surface bubbles. Because the Pt layers deposited with Cs^+^ ions at 2 kV are so sensitive to air exposure, the deposition rate and the resistivity measurements will mainly focus on layers deposited with acceleration voltages of 5, 8, and 16 kV.

### Deposition rates

**Figure 4:**
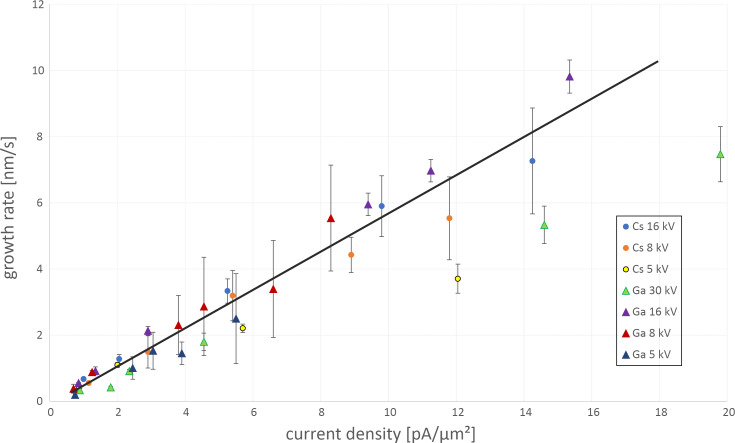
Pt growth rate vs ion current density for different acceleration voltages of Cs^+^ and Ga^+^ ions. As a guide to the eye, a line for the average growth rate is shown. With increasing current density, the growth rate increases. Only for Ga^+^ ions at 30 kV and Cs^+^ ions at 5 kV, the growth rates at higher current densities are much lower than the average.

Deposition rates of Ga^+^ and Cs^+^ FIBID-Pt were measured using the dimensions of the cross sections determined via FIB cut and SEM imaging. The deposition rates of Pt on Si (shown in Figure 4) increase with increasing ion current density. This applies to almost all voltages for Cs^+^ as well as for Ga^+^ ions. Only for Ga at 30 kV and a current density above 6 pA·μm^−2^, the growth rate is lower than those for all other beam parameters. This is due to the fact that the sputter rate increases with beam current while the deposition rate is saturated due to the limited gas flow, which leads to an overall lower increase in growth rate with increasing current density. This agrees with previous findings for Ga^+^ FIBID-Pt [25]. Also, for Cs^+^ at 5 kV, the deposition rate is lower. A possible explanation might be the broader beam diameter, which increases for lower acceleration voltages (*<*5 kV) and higher ion beam currents (*>*200 pA). This leads to a lower current density in a beam spot compared to the current density at higher voltages for the same ion beam current. Thus, while the overall current density (ion beam current per unit area of the pattern) is the same, the local density is lower, which could result in a lower growth rate. In the future, further measurements could be done with finer variations of the ion beam current starting at 100 pA and 5 kV to verify this assumption.

### Composition and microstructure

**Figure 5:**
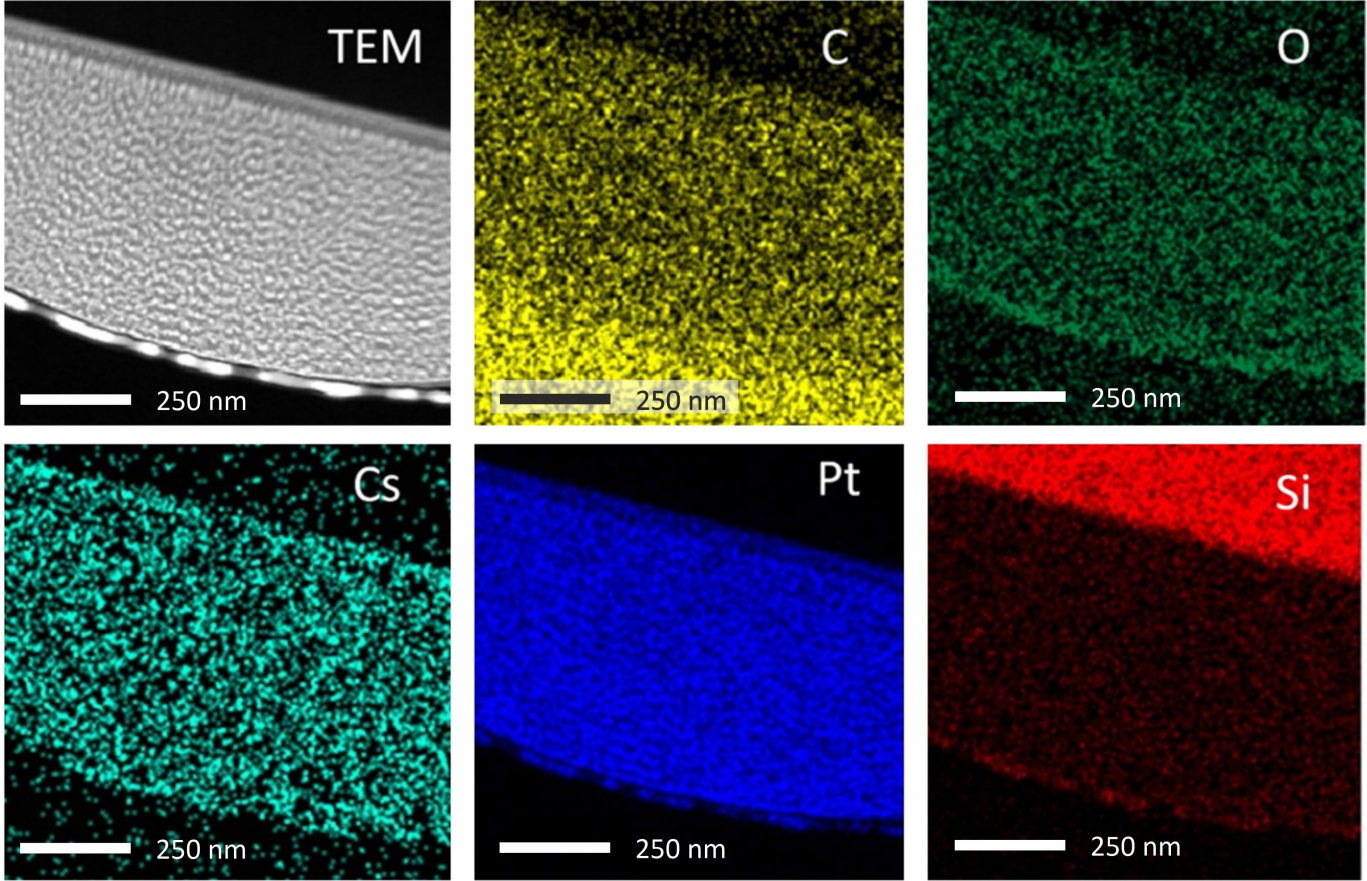
TEM-EDS mapping for the Pt deposit induced with 16 kV 54 pA Cs^+^.

**Figure 6:**
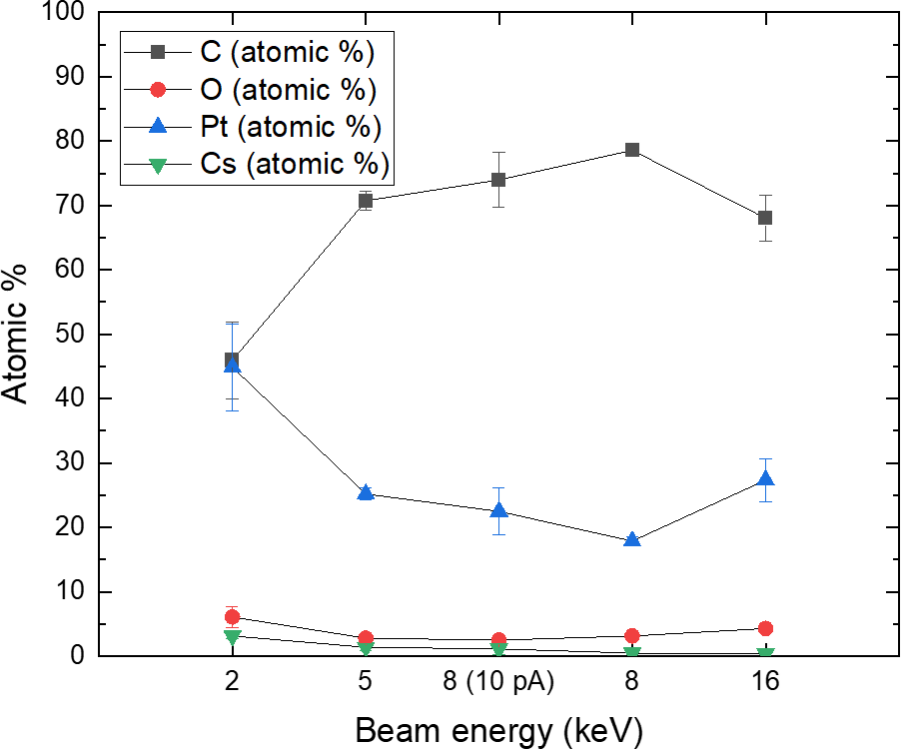
Cs^+^-induced Pt composition data. Other than for the 8 kV 10 pA data, 54 pA was used for deposition with different beam energies.

Compositional data of the Cs^+^ FIBID-Pt were calculated from the data provided by TEM-EDS analysis. An exemplary EDS map for 16 kV 54 pA Cs^+^ FIBID-Pt is shown in Figure 5. The Si-rich region shown as the red area in the upper-right corner of the Si map corresponds to the Si substrate. Before the TEM sample preparation process, a C layer was deposited on top of the Cs^+^ FIBID-Pt deposits using focused electron beam-induced deposition (FEBID). Therefore, a C-rich region exists in the lower-left corner of the C map. EDS spectra were taken at five points within the bulk deposit and then averaged to determine the chemical composition. Figure 6 displays these compositional data for C, O, Pt, and Cs with standard deviations of the average as uncertainties.

From the data shown in Figure 6, it follows that the Pt deposits created at 8 kV and 10 pA Cs^+^ result in an atomic abundance ratio of C/O/Pt/Cs = 74:2.5:23:1. Compared to Rb^+^ FIBID-Pt of similar beam energy, current, and ion dose (8.5 kV 7 pA Rb+ delivers C/O/Pt/Rb = 25:20:49:5, and 8.0 kV 8.5 pA Ga+ delivers C/O/Pt/Ga = 22:14:37:27) presented in [11], Cs^+^ FIBID-Pt thus is found to have lower O, Pt, and Cs content and higher C content. This C content could be partially affected by the FEBID-C protective layer for the TEM sample. However, the EDS data were reported without adjustment to the C% data because the 2 kV 54 pA Cs^+^ FIBID-Pt included in Figure 6 exhibits much lower C% despite being on the same TEM lamella as the other deposits. In addition, very little Si% appears within the deposit area, which demonstrates the location sensitivity of TEM-EDS. This location sensitivity makes it likely that the FEBID-C protective layer is not the main contributor to the higher C% of Cs^+^ FIBID-Pt. The composition of the Cs^+^ FIBID-Pt stays mostly constant for beam energies above 2 kV. The 2 kV Cs^+^ FIBID, being closer to Rb^+^ FIBID-Pt in Pt% and C%, is an outlier compared to the FIBID under higher beam energies. The cause for the higher C% in Cs^+^ FIBID-Pt remains unclear to the authors at this stage, however.

**Figure 7:**
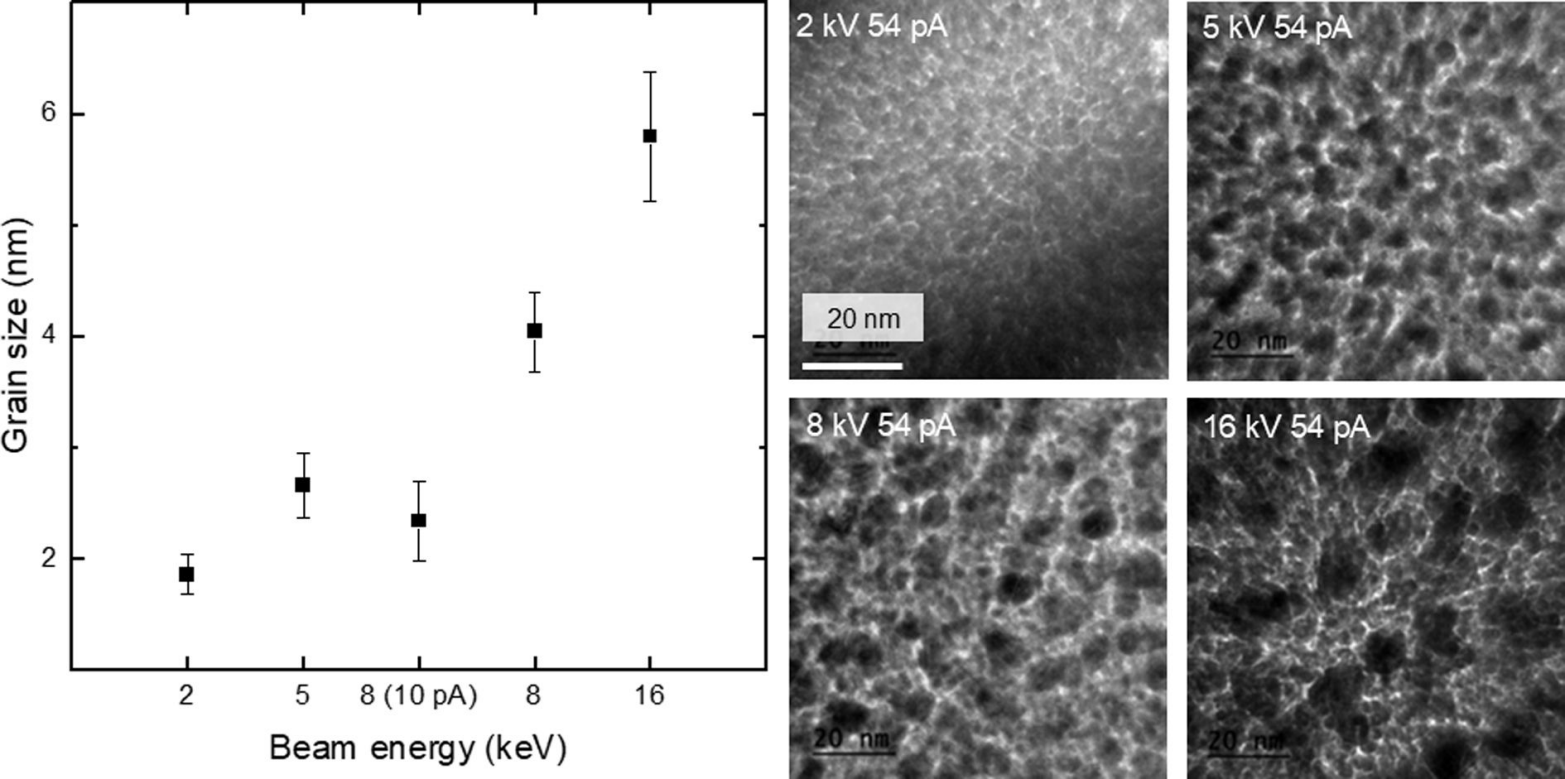
Bright-field images of Cs^+^-induced Pt deposit and Pt grain size data for different acceleration voltages.

As with Rb^+^ and Ga^+^, Cs^+^ FIBID-Pt also contains crystalline Pt grains embedded in a C-rich matrix, as shown in Figure 7. These bright-field TEM images were used for grain diameter measurements in the same way as done in [11]. In short, bright-field TEM images similar to those shown in Figure 7, but taken at two times higher magnification, were analyzed using the particle analyzer option provided by the image processing software ImageJ [26]. The Pt grains appear darker than the C matrix in the TEM images, thus allowing the grains to be counted by ImageJ. This software also provides the area of each grain counted, which we convert to an effective diameter by equating the area to that of a circular disk. Figure 7 shows that the average grain diameter grows from 1.9 to 5.8 nm when the beam energy increases from 2 to 16 kV. The increase in grain size with beam energy is visually obvious from the bright-field images shown in Figure 7. Previously, De Teresa et al. reported a 3.2 ± 0.8 nm grain diameter for 5 kV Ga^+^ FIBID-Pt [25], which is similar to the 2.7 ± 0.3 nm grain diameter for 5 kV Cs^+^ FIBID-Pt. The grain diameter of 5.8 nm at 16 kV Cs^+^ FIBID-Pt is similar to the diameters of 8.5 kV Rb^+^ and 8 kV Ga^+^ FIBID-Pt. Thus, lower-energy Cs^+^ creates deposits with finer grains. It is worth noting that the growth in grain diameter does not correlate with the EDS data, in which the composition remains nearly constant for above 2 kV Cs^+^ FIBID-Pt.

### Deposit resistivity

**Figure 8:**
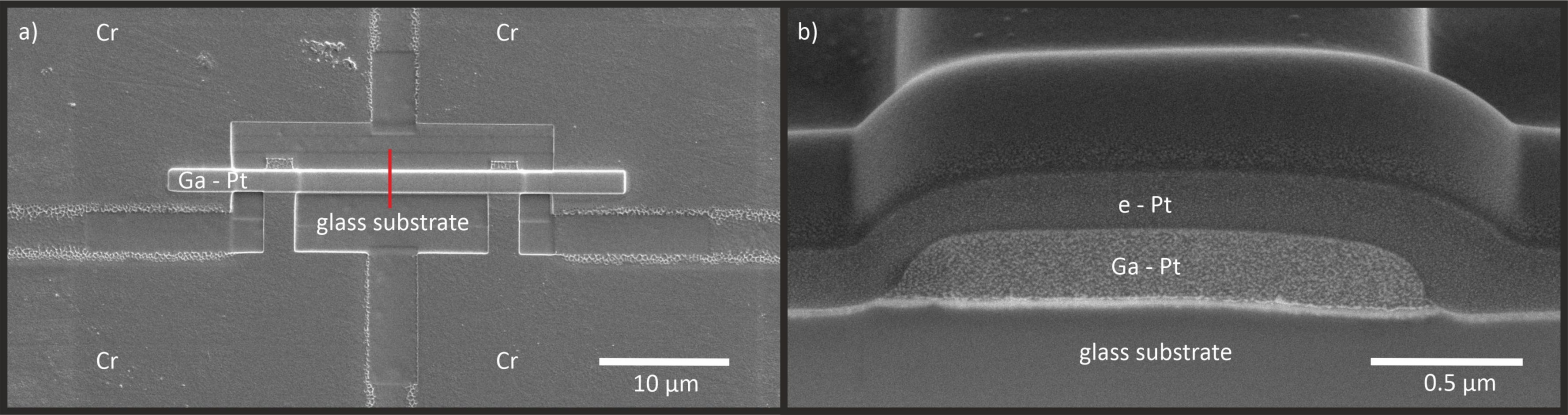
(a) Structure for the resistivity measurements consisting of four Cr electrodes and the 35 μm by 1.5 μm Ga-induced Pt deposit layer. The red line indicates the position of the cross section. (b) SEM image of this cross section. Before the FIB cut for the cross section measurement was done, an additional Pt layer was deposited with FEBID for protection.

The electrical resistivity was measured with a four-point probe setup as shown in Figure 8a. The electric current was pushed trough the deposited layer using the two upper contacts, while the resulting voltage was measured between the two lower contacts to evaluate the resistivity *R* in the same way as in [11]. After these measurements were done, the area *A* of the cross section was determined with a FIB cut and SEM image (see Figure 8b). The length *l* between the contacts was about 14.6 μm, so the electrical resistivity can be calculated with *R* = ρ*l*/*A* for each layer.

**Figure 9:**
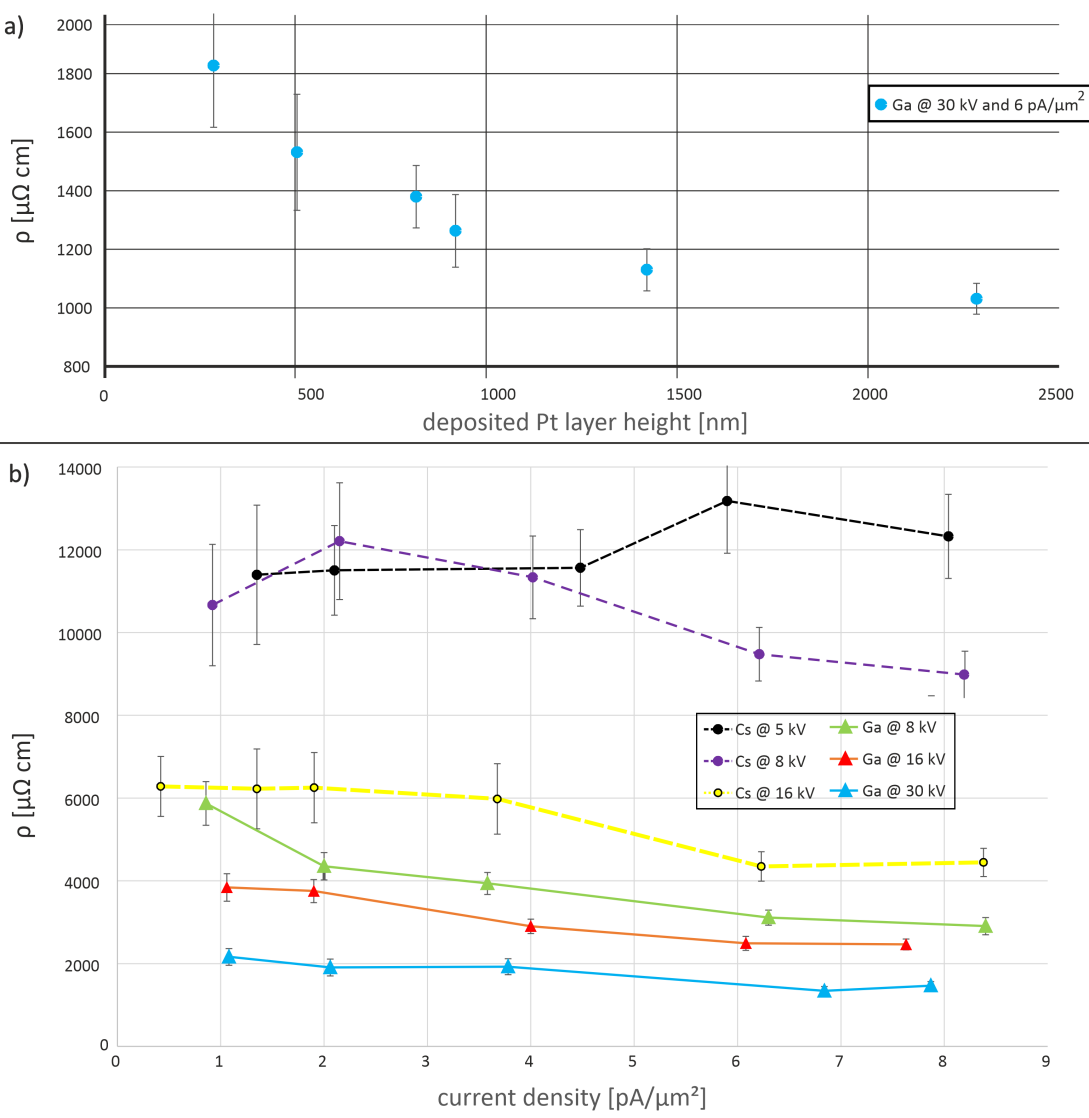
(a) Resistivity of Ga^+^-induced Pt deposits using the same current density and acceleration voltage. Only the layer thickness was varied. Thicker layers have a lower electrical resistivity. (b) Resistivity vs current density for different acceleration voltages for Ga^+^ and Cs^+^ ions. With increasing acceleration voltage, the electrical resistivity decreases. Furthermore, the Ga^+^ FIBID-Pt has a lower resistivity than the Cs^+^ FIBID-Pt.

For each beam setting, the deposition time was calculated using the growth rate shown in Figure 4 in order to deposit Pt layers with a thickness of 1 μm. Because here the substrate is glass and not Si, charging effects can occur. In addition, the SE yields of amorphous SiO_2_ and crystalline Si are different. Therefore the actual deposition rates and the estimated and real layer thickness differ. Overall, they vary from 300 to 1200 nm. Although in theory, the area of the cross section should not have an influence on the calculation of the specific resistivity, De Teresa et al. showed that there can be an impact [25,27]. Therefore, preliminary measurements were carried out to study the thickness dependence of the resistivity. With the Ga^+^ FIB, Pt layers were deposited with an ion current density of 6 pA·μm^−2^ at a voltage of 30 kV. Only the deposition time and thus the layer thickness was varied. The results can be seen in Figure 9a. We find that layers thicker than 1 μm have a lower electrical resistivity compared to thinner ones by up to a factor of two in the range covered. We conclude that for our conditions, layer thickness does indeed have an influence on the resistivity of Ga^+^ FIBID-Pt deposits.

Figure 9b contains measured resistivity results for Ga^+^- and Cs^+^-induced Pt deposits for different current densities and acceleration voltages. The resistivity of Cs^+^ FIBID-Pt decreases with increasing primary ion beam energy. For 16 and 8 kV Cs^+^ FIBID-Pt, the resistivity also decreases with higher Cs^+^ ion current density. Only for 5 kV, the resistivity increases at higher beam currents. For all acceleration voltages used with Ga^+^ ions, that is, 30, 16, and 8 kV, the electrical resistivity decreases with increasing current density. At all voltages, the resistivity of Cs^+^ FIBID-Pt is higher than that of Ga^+^ FIBID-Pt. Only Pt deposited with Cs^+^ ions at 16 kV has almost the same value as Ga^+^ FIBID-Pt. The other voltages create layers with at least three times higher specific resistivity values.

The decrease in resistivity for Ga^+^ FIBID-Pt with increasing ion current has already been reported [28] and is now verified in the measurements reported here. In particular, the Cs^+^ FIBID-Pt layers deposited with a current density below 6 pA·μm^−2^ have a thickness below 1 μm. As shown in Figure 9a, this may lead to a higher electrical resistivity. The changes in resistivity are not immediately obvious from the chemical composition of the deposits. Speculation based on the microstructure provides a probable cause for this decreasing resistivity vs beam energy. Figure 7 shows that the average grain diameter grows from 1.9 to 5.8 nm when the beam energy increases from 2 to 16 kV. With larger grain diameters, the Pt-rich particles have larger surface areas in close contact. These larger surface areas help the current to flow more easily between the Pt deposits. Therefore, the deposit resistivity decreases despite a similar chemical composition. However, it is unclear why Cs^+^ FIBID-Pt would have a higher resistivity than Ga^+^ FIBID-Pt under similar beam conditions since the Cs^+^ FIBID was observed to have the lowest O%, which should lead to a smaller resistivity. So this might be a combined effect of chemical composition and grain size. However, the acceleration voltage and the ion species seem to play a more dominant role than the ion current density for the electrical resistivity. Further studies are required to fully investigate the resistivity–microstructure dependency for FIBID-Pt.

To compare the Cs^+^ and Rb^+^ FIBID, Pt layers were deposited with the same beam settings, namely, a very low current density of about 0.2 pA·μm^−2^ at a voltage of 8 kV. The electrical resistivity of Cs^+^ FIBID-Pt is (3.2 ± 0.4) × 10^4^ μΩ·cm, which is about four times lower compared to Rb^+^ FIBID-Pt with a resistivity of about (12 ± 4) × 10^4^ μΩ·cm [11]. Also, one Pt layer was deposited with Cs^+^ ions at 2 kV and a beam current density of 3.76 pA·μm^−2^. Here the resistivity is (3.4 ± 0.3) × 10^4^ μΩ·cm, which is three to five times higher compared to other Cs^+^ induced Pt layers. We conclude that alkali metal ion beams operating at low current densities and acceleration voltages lead to high electrical resistivity of the Pt deposits.

## Conclusion

In this paper, we show that it is possible to deposit Pt with a Cs^+^ FIB. Growth rates were measured for Ga^+^ and Cs^+^ ions at different acceleration voltages. The rate mainly increases linearly with ion current density. Pt layers deposited with Cs^+^ ions at 2 and 5 kV react with air and form bubbles. This similarity in the occurrence of surface bubbles between Cs^+^ and Rb^+^ may reveal a characteristic phenomenon of alkali metal ion-induced deposition. The electrical resistivity of the deposited Pt decreases with increasing acceleration voltage and is mostly independent of the ion current density. The Cs^+^ FIBID-Pt has resistivity values between those of Ga^+^ and Rb^+^ FIBID-Pt, while having a lower Pt content. Lower current densities and acceleration voltages were found to lead to a higher electrical resistivity.

## Appendix

Table 2 contains the complete set of parameters of the FIBID for the growth rate measurements.

**Table 2:** Complete set of parameters of the FIBID for the growth rate measurements.

Ion	Acceleration voltage [kV]	Ion current [pA]	Current density [pA·μm^−2^]	Step size [nm]	Layer height [nm]	Growth rate [nm/s]	Volume per dose [μm^3^/nC]

Cs	16	20	1.00	80	101	0.67	0.67
	16	41	2.05	100	192	1.28	0.62
	16	105	5.25	100	500	3.33	0.63
	16	196	9.80	100	885	5.90	0.60
	16	285	14.25	100	1090	7.27	0.51
	8	21	1.15	100	83	0.55	0.48
	8	62	2.90	100	224	1.49	0.51
	8	130	5.40	100	479	3.19	0.59
	8	220	8.90	150	664	4.43	0.50
	8	300	11.80	150	830	5.53	0.47
	5	17	0.85	100	57	0.38	0.45
	5	40	2.00	100	165	1.10	0.55
	5	114	5.70	200	331	2.21	0.39
	5	241	12.05	200	556	3.71	0.31

Ga	30	17.8	0.89	24.5	58	0.39	0.43
	30	36	1.80	32.5	122	0.81	0.45
	30	47	2.35	40	157	1.05	0.45
	30	91	4.55	52.5	309	2.06	0.45
	30	292	14.60	95	885	5.90	0.40
	30	396	19.80	132.5	1225	8.17	0.41
	16	17	0.85	40	83	0.55	0.65
	16	27	1.35	50	138	0.92	0.68
	16	58	2.90	67.5	320	2.13	0.74
	16	188	9.40	187.5	893	5.95	0.63
	16	225	11.25	250	1046	6.97	0.62
	16	307	15.35	250	1473	9.82	0.64
	8	14	0.70	55	57	0.38	0.54
	8	25	1.25	80	132	0.88	0.70
	8	76	3.80	145	346	2.31	0.61
	8	91	4.55	210	430	2.87	0.63
	8	132	6.60	220	509	3.39	0.51
	8	166	8.30	295	831	5.54	0.67
	5	15	0.75	100	29	0.19	0.26
	5	49	2.45	190	151	1.01	0.41
	5	61	3.05	232.5	229	1.53	0.50
	5	78	3.90	250	218	1.45	0.37
	5	110	5.50	397.5	375	2.50	0.45

## Data Availability

Data generated and analyzed during this study is available from the corresponding author upon reasonable request.
